# Silencing of E2F3 suppresses tumor growth of Her2^+^ breast cancer cells by restricting mitosis

**DOI:** 10.18632/oncotarget.5686

**Published:** 2015-10-26

**Authors:** Miyoung Lee, Gabriela Oprea-Ilies, Harold I. Saavedra

**Affiliations:** ^1^ Department of Radiation Oncology, Emory University School of Medicine, Atlanta, GA 30322, USA; ^2^ Department of Pathology and Laboratory Medicine, Emory University School of Medicine, Grady Memorial Hospital, Atlanta, GA 30303, USA; ^3^ Department of Basic Sciences, Pharmacology Division, Ponce Health Sciences University, Ponce Research Institute, Ponce, P.R. 00716-2348, USA

**Keywords:** E2F3, Her2^+^ breast cancer, centrosome amplification, mitosis, xenograft mammary tumor model

## Abstract

The E2F transcriptional activators E2F1, E2F2 and E2F3a regulate many important cellular processes, including DNA replication, apoptosis and centrosome duplication. Previously, we demonstrated that silencing E2F1 or E2F3 suppresses centrosome amplification (CA) and chromosome instability (CIN) in Her2^+^ breast cancer cells without markedly altering proliferation. However, it is unknown whether and how silencing a single E2F activator, E2F3, affects malignancy of human breast cancer cells. Thus, we injected HCC1954 Her2^+^ breast cancer cells silenced for E2F3 into mammary fat pads of immunodeficient mice and demonstrated that loss of E2F3 retards tumor growth. Surprisingly, silencing of E2F3 led to significant reductions in mitotic indices relative to vector controls, while the percentage of cells undergoing S phase were not affected. Nek2 is a mitotic kinase commonly upregulated in breast cancers and a critical regulator of Cdk4- or E2F- mediated CA. In this report, we found that Nek2 overexpression rescued back the CA caused by silencing of shE2F3. However, the effects of Nek2 overexpression in affecting tumor growth rates of shE2F3 and shE2F3; GFP cells were inconclusive. Taken together, our results indicate that E2F3 silencing decreases mammary tumor growth by reducing percentage of cells undergoing mitosis.

## INTRODUCTION

The E2F transcription factors are master regulators of multiple biological functions, such as DNA replication, DNA repair, apoptosis, and centrosome duplication [1–8]. Because of their versatility, E2Fs are frequently deregulated and altered in most human cancers through multiple molecular mechanisms. For example, overstimulation of the G_1/_S phase cyclin/Cdks complexes hyper phosphorylates and inactivates the Rb family of tumor suppressors [9, 10]. E2F's are also overexpressed, exemplified by E2F3 up regulation in various cancers, including breast cancers [11–17]. Consistent with these observations, we showed that all three E2F activators, E2F1, E2F2 and E2F3a, are highly deregulated in Her2^+^ breast cancer cells [18] while Wu *et al*. demonstrated E2F3a up-regulation in primary mouse or human tumors with amplified Her2/ERBB2 [17]. In fact, deregulated expression of the E2Fs along with Cyclin A in breast cancers negatively influences disease free survival [19]. Recently, mouse models demonstrated that the E2F activators are required in mammary tumor initiation, since ablation of E2F1 and E2F3 suppressed Her2/Neu and Myc-induced mammary tumorigenesis [17, 20, 21]. However, it is unknown whether and how interfering with E2F function influences tumor progression of human breast cancer cells, particularly of Her2^+^ (Her2^+^ER^−^PR^−^) tumors, which is one of the two most aggressive and poorly prognostic breast cancer subtypes [22]. In this report, we used an orthotopic model of Her2^+^ breast cancer to address how manipulation of E2F3 levels influences mammary tumorigenesis.

Various mechanisms may explain how the E2F activators drive mammary tumorigenesis. The E2F activators were initially distinguished from repressors based on their ability to induce S phase entry [23–25], therefore a likely mechanism for E2F mediated mammary tumorigenesis is deregulation of DNA replication [26, 27]. In particular, E2F3 is central to DNA replication, since either expression of E2F3a or E2F3b transcripts is sufficient to drive E2F target genes and maintain cell proliferation in the absence of the other two activators, E2F1 and E2F2 [5]. In addition, conditional knockdown of E2F3 in the mammary epithelium of ERBB2 transgenic mice resulted in significant decreases in percentages of proliferating cells, while ablation of E2F1 had no effect [17]. Although the regulation of S phase is a canonical function of the E2Fs activators, it is unclear if and how they regulate mitosis. By using expression microarrays, Ishida *et al* identified E2F-regulated genes in mouse embryo fibroblast (MEF) [8], including a plethora of genes that control DNA replication, and a smaller number of genes involved in mitosis, including Cdc2 and cyclin B1. Overexpression of mitotic genes was detected upon zinc-induced E2F3 overexpression in the Rat-1 rat non-tumorigenic fibroblasts [28]. More recent reports suggest that E2Fs control entry into G_2_/M by regulating levels of Aurora A [29], Emi and the polo kinases [30]. Our own work demonstrated that stable silencing of E2F3 resulted in lower levels of the Nek2 mitotic kinase and delayed cytokinesis and that introduction of Nek2 reversed that delay, suggesting that Nek2 is an important regulator of cytokinesis downstream of E2F3 [18].

A second mechanism by which E2Fs may influence mammary tumorigenesis is through centrosome amplification (CA), a malignant phenotype where cells acquire supranumerary centrosomes to generate multipolar mitosis and aneuploidy, one of the landmarks of chromosome instability (CIN) [31–33]. CA is postulated to influence mammary cancer initiation and progression; for example, most breast adenocarcinomas display CA [34–36] and elevated percentages of CA are already present in pre-malignant mammary tumor lesions [35, 36], where it is in part driven by oncogenic K-Ras^G12D^ [37]. Although the role of CA and CIN as initiators of breast tumor formation remains to be tested experimentally, their presence in poor prognosis breast tumors, including Her2^+^ and Her2^−^ER^−^PR^−^ (triple-negative), correlates with their aggressive behavior, marked by increased proliferation indexes, high nuclear grades, invasion, metastasis and poor survival [32, 35, 36, 38–42]. Our group demonstrated that silencing E2F1 or E2F3 suppressed CA/CIN in Her2^+^ breast cancer, while their overexpression in mammary epithelial cells triggered CA/CIN that respectively correlated with decreases or increases in Nek2 protein levels. We also found that Nek2 acts downstream of the Rb pathway, since Nek2 overexpression rescues back CA/CIN in Her2^+^ breast cancer cells silenced for Cdk4 [43] or E2F3 [18]. A third major mechanism by which the E2Fs may influence mammary tumorigenesis is by signaling apoptosis. For example, E2F1 and E2F3 mediate apoptosis in the central nervous system of Rb-null embryos [44]. The role of E2F1 in inducing p53-dependent and independent apoptosis has been thoroughly reviewed [45].

To investigate the involvement of E2F3 in mammary tumor progression, we used stable silencing of E2F3 in a human Her2^+^ breast cancer cell line and an orthotopic xenograft mouse model. We chose the HCC1954 cell line based on the evidence of successful tumor formation in a xenograft mouse model [46] and because it is a highly malignant breast cancer cell line displaying CA/CIN, radiation and Herceptin resistance [47, 48]. We found mammary tumor progression was significantly decreased upon E2F3 silencing. Mechanistically, shE2F3 expression resulted in significant decreases in CA and mitosis. Surprisingly, although Nek2 overexpression in cells knocked down for E2F3 increased percentages of CA in tumors, its effects regarding tumor growth are inconclusive, since it did not significantly affect mammary tumor progression in shE2F3 cells, but led to bigger tumors at early time points relative to shE2F3 cells expressing GFP.

## RESULTS

### E2F3 knockdown decreases tumor growth

**Figure 1:**
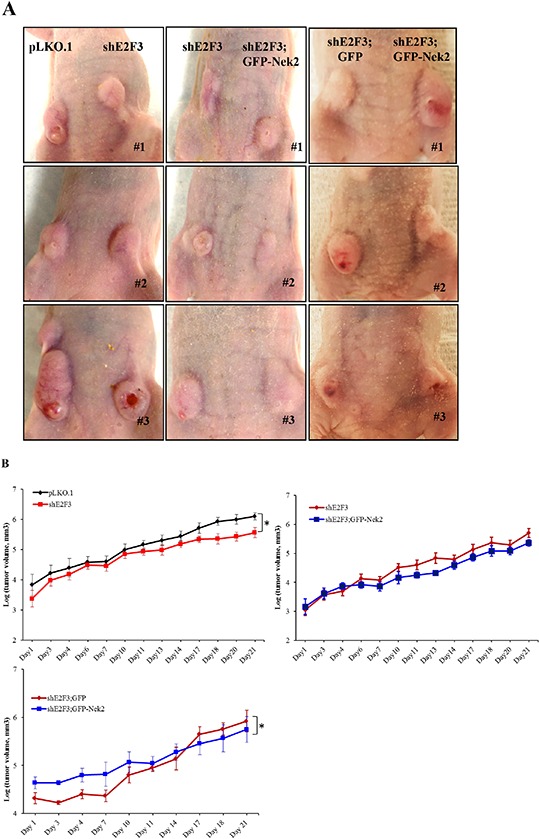

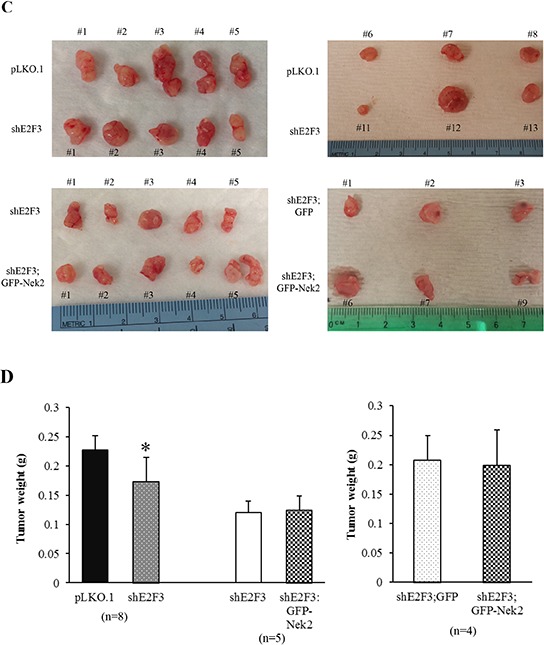

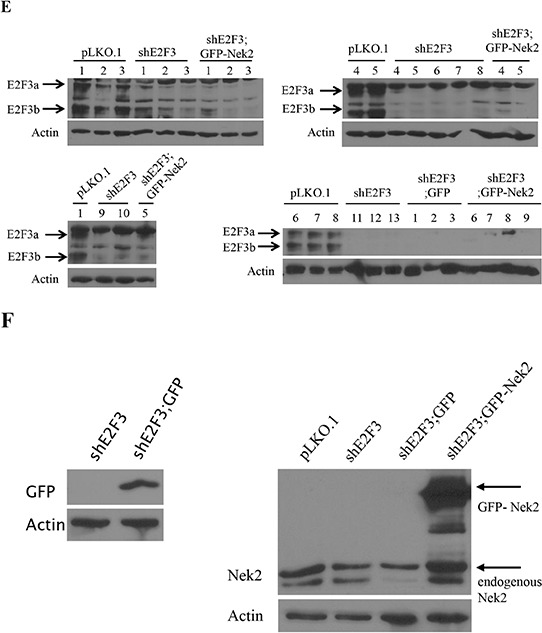
E2F3 knockdown decreases tumor growth and burden of Her2+ HCC1954 cells Tumor growth was monitored starting one week post injection 5–6 times/week and measured for 21 days. **A.** Pictures were taken from mice bearing tumor masses in mammary glands and numbers indicate mouse number. **B.** The tumor growth rate was plotted over time and compared between control pLKO.1 vs. shE2F3 (*N* = 8), shE2F3 vs. shE2F3; GFP-Nek2 and shE2F3;GFP vs. shE2F3; GFP-Nek2 for 21 days after detection of tumor masses. Tumor masses were extirpated. **C.** and weighted **D.** Western blots were performed to detect changes in protein level of E2F3 from tumor masses. **E.** or GFP, Nek2 and GFP-Nek2 from cell lines **F.**

The goal of this series of experiments is to establish whether silencing E2F3 with or without the introduction of Nek2 modifies tumor progression of HCC1954 breast cancer cells *in vivo*. We analyzed three sets of mice. In the first set, mice were injected in the hind, left mammary gland #9 with HCC1954 cells stably expressing pLKO.1 empty vector and the right #4 gland with cells stably expressing shE2F3. The second set of mice were injected in the left mammary gland #9 with cells expressing shE2F3 and with cells expressing shE2F3; GFP-Nek2 in the right mammary gland #4. A third group was injected with shE2F3; GFP on the left mammary gland #9 and with shE2F3; GFP-Nek2 on the right #4. Tumor masses were palpable one week after injection, and at that point we started measuring tumor growth 5–6 times/week for three weeks (Figure 1A showed tumors at the time of sacrifice). As seen in Figure 1B and Table 1, tumors grew significantly slower in HCC1954 cells silenced for E2F3 compared to vector control (pLKO.1). Tumors were extirpated (Figure 1C) and tumor masses were weighed. Tumors expressing shE2F3 were significantly lighter than pLKO.1 control, indicative of suppressed tumor burden (Figure 1D).

**Table 1:** Estimated tumor growth rate by experiment groups

Group	Mean (Std.Err)	*p*-value*
pLKO.1 (*n* = 8)	0.114 (0.005)	ref
shE2F3 (*n* = 8)	0.1 (0.005)	0.031
shE2F3; GFP (*n* = 4)	0.091 (0.005)	ref
shE2F3; GFP-Nek2 (*n* = 4)	0.057 (0.005)	<0.001
shE2F3 (*n* = 5)	0.11 (0.004)	ref
shE2F3;GFP-Nek2 (*n* = 5)	0.1 (0.004)	0.107

* Mixed effects model was implemented to estimate and compare the growth rate among three cell line groups using the SAS statistical package v9.3 with a significant level of 0.05.

A controversial issue is whether the high CIN triggered by CA is oncogenic or tumor suppressive [49–51]. In a previous publication, we showed that HCC1954 cells stably silenced for E2F3 displayed lower percentages of CA and CIN relative to control and expression of GFP-Nek2 re-established high levels of CA in Her2^+^ cells expressing shE2F3 [18]. Next, we tested whether CA and CIN affect tumor growth, since HCC1954 cells display inactivating mutations in p53 [52] and can tolerate binucleation, an important intermediate to polyploidy [18, 43]. In addition, loss of p53 function allows maintenance of polyploid cells in some cell lines [53, 54]. Overexpression of Nek2 (full length of Nek2 cDNA was subcloned into pMONO-Hygro-GFP vector [18]), did not change the growth rates or average tumor mass of HCC1954/shE2F3 cells (Figure 1B, 1C-D). However, in an independent experiment that tested whether there were differences in grown rates between shE2F3; GFP and shE2F3; GFP-Nek2, the overall tumor growth rate between shE2F3; GFP and shE2F3; GFP-Nek2 was statistically significant due to larger tumor volumes in the shE2F3; GFP-Nek2 mice between days 1 and 10 and no differences were found at later time points (Figure 1B, Table 1). Similar to results from the first experiment comparing shE2F3 and shE2F3; GFP-Nek2, no differences were found in tumor weight between shE2F3; GFP and shE2F3; GFP-Nek2 (Figure 1D).

Proteins were extracted from tumors and western blot analyses revealed that E2F3a and E2F3b remained significantly down regulated in most shE2F3 tumors relative to pLKO.1 controls (Figure 1E). Western blot detected GFP in shE2F3; GFP cell line, confirming the generation of GFP expressing shE2F3 cells (Figure 1F). Nek2 western blot was performed in HCC1954 cell lines from each group and expression of endogenous Nek2 was detected in all samples and the GFP-Nek2 fusion protein was detected only in cells expressing GFP-Nek2. Overall, these experiments demonstrated that silencing of E2F3 suppresses tumor growth and tumor mass of HCC1954 cells, while effects of GFP-Nek2 overexpression in HCC1954 cells silenced for E2F3 are inconclusive.

### E2F3 knockdown restricts percentage of Her2^+^ breast tumor cells undergoing mitosis

**Figure 2:**
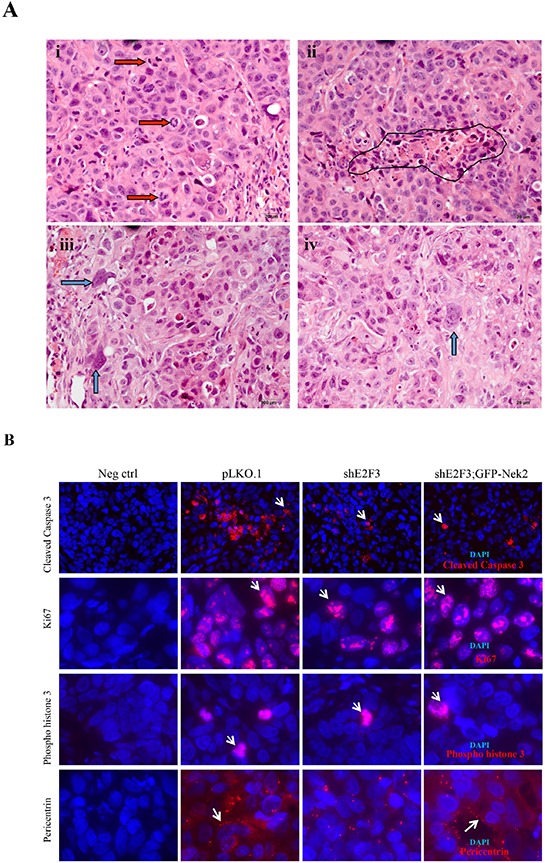

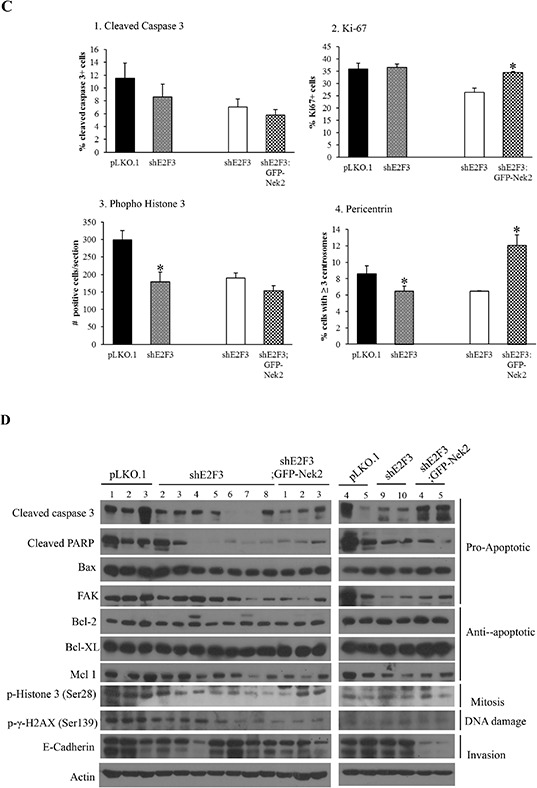

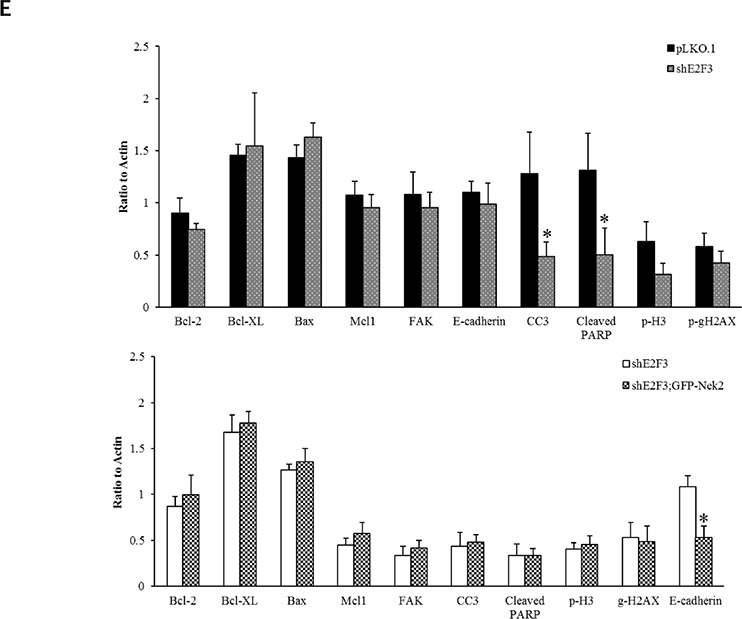
E2F3 knockdown restricts percentage of Her2+ breast tumor cells undergoing mitosis **A.** Representative H&E slides showing frequent mitosis (red arrows), punctate necrosis (black circle), (iii) anaplastic and high grade nuclei (green arrows), (i, ii and iii from pLKO.1, iv from shE2F3;GFP-Nek2 mouse **B.** Immunofluorescence (IF) was performed on paraffin embedded tumor sections to detect apoptosis (cleaved caspase 3), cell proliferation (Ki67), mitosis (phospho histone 3) and centrosome amplification (pericentrin); magnification: cleaved caspase 3 (20X), Ki67, Phospho histone 3, pericentrin (63x). **C.** Quantification of IF (N = 5, *p ≤ 0.05). The percentage of cells was calculated electronically by measuring the percentage of cleaved caspase 3 cells from the ratio of Red/DAPI, and the ratio of Red/DAPI for Ki67. Positive stained cells were counted on the entire sections for phospho histone-3 and centrosome amplification by counting cells with ≥ 3 centrosomes using pericentrin. Protein lysates were prepared from tumor masses and 15 μg of protein was analyzed with Western blots. **D.** to detect changes of protein level in various cellular processes and their levels were quantified. **E.** as pLKO.1 vs. shE2F3 and shE2F3 vs. shE2F3; GFP-Nek2. *N* = 5 for Bcl2, Bcl-XL, Bax, Mcl1, FAK and E-Cadherin for pLKO.1 vs. shE2F3 and shE2F3 vs. shE2F3; GFP-Nek2. *N* = 5 for cleaved Caspase-3, Cleaved PARP, phospho-histone H3 and Phospho-γ-H2AX for shE2F3 vs. shE2F3; GFP-Nek2 and *N* = 8 for pLKO.1 vs. shE2F3 (*p ≤ 0.05)

To identify mechanisms responsible for the reduced tumor growth of cells silenced for E2F3, we first performed histopathology of tumor sections. Histopathologic examination of H&E stained slides showed all tumors to have a high grade, with no tubule formation, high nuclear grade and numerous, up to 52 mitoses/10 high power field–hpf- (Figure 2A, indicated by red arrows and Table 2). High nuclear grade was accompanied by anaplastic nuclei with irregular nuclear contour, and atypical, hyperchromatic or open chromatin patterns (green arrows). Tumors from the control group of pLKO.1 *vs* shE2F3 pairs showed measurable necrosis (circled area), extensive, eccentric or para-medially located, comedo-like measuring in average 2.3 mm sq, with a median of 2.5 mm sq. The necrotic area represented on average 13.98% of the tumor area. The average mitosis rate was 28.4/10 hpf. In the shE2F3 group of pLKO.1 *vs* shE2F3 pairs, the necrosis was measurable, extensive, eccentric or para-medially located, comedo-like in half of mice. On average, the necrotic area measured 1.4 mm sq, with a median of 2 mm sq, on average 10.5% of all tumor area. Half of tumors showed rather small, less than 0.1 mm necrosis and single cell necrosis dispersed throughout the tumor. The average mitosis rate was 30/10 hpf. In the shE2F3 group of shE2F3 vs shE2F;GFP-Nek2 pairs, two tumors (#7, 8) showed measurable necrosis (#7 with paramedical and comedo-like and #8 with central and comedo-like) para-medial in average 2.66 mm sq that represented average 8.96% of tumor area of all 5 mice. The average mitosis rate was 38/10 hpf. In the shE2F3; GFP-Nek2 group, only one tumor (shE2F3; GFP-Nek2 #3) showed measurable necrosis, which was extensive para-medially located, comedo-like. It measured 4.0 mm sq and represented 20% of that tumor area, for average of 0.8% for that group. The average mitosis rate was 39.6/10 hpf. In summary, the tumors in the shE2F3; GFP-Nek2 group had the highest mitotic count relative to control or shE2F3 groups. All tumors in the pLKO.1 control group showed measurable, comedo-like necrosis, while only half of tumors in the shE2F3 group and only one tumor in the shE2F3; GFP-Nek2 group had this appearance. Even though there were differences in the averages of some phenotypes described in this paragraph, they were not statistically significant.

**Table 2:** Pathological Analysis on H&E stained Sections

Group	Percentage necrosis/tumor	Necrosis dimension (mm)	Surface, necrosis (mm sq)	Description of necrosis	^1^mits	^2^NC grade	^3^Tubules
pLKO.1_1	22	2 × 1.5	3	^4^Excentric, small foci and dispersed	38	3	3
pLKO.1_2	19	2 × 1.5	3	Excentric, ^5^comedo-like	20	3	3
pLKO.1_3	7.4	2 × 1	2	Excentric, comedo-like	22	3	3
pLKO.1_4	5.7	1 × 1	1	Paramedial, comedo-like	24	3	3
pLKO.1_5	15.8	2.5 × 1	2.5	Paramedial, comedo-like, disperesd	38	3	3
**Ave**	**13.98**		**2.3**		**28.4**		
**SD**	**7.153**		**0.837**		**8.877**		
shE2F3_1	na	Single cell necrosis	Na	Dispersed	38	3	3
shE2F3_2	8.08	2 × 1	2	Excentric, comedo-like, dispersed	24	3	3
shE2F3_3	Na	Single cell necrosis	Na	Dispersed	30	3	3
shE2F3_4	23.8	2.5 × 1	2.5	Excentric, comedo, single cell necrosis	28	3	3
shE2F3_5	20.8	2.5 × 1	2.5	Excentric, comedo-like	30	3	3
**Ave**	**10.536**		**1.4**		**30**		
**SD**	**11.284**		**1.294**		**5.099**		
Group	Percentage necrosis/tumor	Necrosis dimension (mm)	Surface, necrosis	Description of necrosis	^1^mits	^2^NC grade	^3^Tubules
shE2F3_6	Na	Single cell necrosis	Na	Dispersed	42	3	3
shE2F3_7	7.9	1.5 × 0.5, 0.75 × 0.75	1.3	Paramedial, comedo-like	38	3	3
shE2F3_8	36.9	4 × 3	12	Central, comedo-like	28	3	3
shE2F3_9	Na	Single cell necrosis	na	Dispersed	38	3	3
shE2F3_10	Na	Single cell necrosis	na	Dispersed	44	3	3
**Ave**	**8.96**		**2.66**		**38**		
**SD**	**15.989**		**5.251**		**6.164**		
shE2F3;GFP-Nek2_1	Na	Single cell necrosis	Na	Dispersed	34	3	3
shE2F3;GFP-Nek2_2	Na	Single cell necrosis	Na	Dispersed	34	3	3
shE2F3;GFP-Nek2_3	20.77	2 × 2	4	Central, Single cell necrosis	52	3	3
shE2F3;GFP-Nek2_4	Na	Single cell necrosis	Na	Single cell nerosis	36	3	3
shE2F3;GFP-Nek2_5	Na	Single cell necrosis	Na	Not significant	42	3	3
**Ave**	**4.154**		**0.8**		**39.6**		
**SD**	**9.289**		**1.789**		**7.668**		

1 =number of mitoses per 10 high power fields

2 = nucleus grade (3 is the highest nuclear grade)

3 =denote a grade of tumor that lost the ability to make tubules/glands (3 is the highest)

4 Exc=excentric

5 Comedo-like=denotes and are of expansive

*denotes multiple tumors, pLKO.1 (1~5) and shE2F3 (1~5) are pair and shE2F3 (6–10) and shE2F3;GFP-Nek2 (1~5) are pair.

To define molecular mechanisms responsible for slower growth of shE2F3 tumors, we measured apoptosis, cell proliferation and mitotic index by immunofluorescence staining (IF) of sections probed with cleaved caspase-3, Ki67 and phospho histone 3, respectively (Figure 2B and 2C). E2F3 knockdown decreased the percentage of cells expressing phospho-histone 3 compared to control, suggestive of reduced number of mitotic cells in tumors. Because there were differences in tumor volumes at initial time points between shE2F3; GFP and shE2F3; GFP-Nek2 and none between shE2F3 and shE2F3; GFP-Nek2 (Figure 1), the immunofluorescence results comparing shE2F3 and shE2F3; GFP-Nek2 presented here have to be interpreted conservatively, since we did not perform such analysis comparing shE2F3; GFP and shE2F3; GFP-Nek2. Nevertheless, phenotypes modified by GFP-Nek2 in shE2F3 cells were high CA, restored to levels even higher than those observed in controls and higher percentage of proliferating cells relative to shE2F3 cells. This later result strengthens our observations that Nek2 is a critical driver of CA/CIN downstream of E2F3 and represents the first *in vivo* evidence that Nek2 induces CA in tumors. Overall, these data demonstrate that E2F3 knockdown impairs several important cellular processes, such as centrosome amplification and mitosis; and the reductions in percentages of cells undergoing mitosis correlates with impaired tumor growth. In contrast, increasing CA in shE2F3 cells by overexpressing GFP-Nek2 does not affect the number of cells undergoing mitosis or cell death, but affects proliferation in tumors.

To confirm these findings, proteins were extracted from tumors for western blot analysis (Figure 2D and Supplementary Figure S1). To determine if silencing E2F3 and overexpression of GFP-Nek2 in shE2F3 cells affected the expression of apoptotic markers [55], FAK, cleaved PARP, cleaved caspase-3 and Bax levels were investigated (Figure 2D and 2E), while only a subset of markers (cleaved PARP, cleaved caspase-3, phospho-Histone H3 and γ-H2AX) was investigated in shE2F3; GFP and shE2F3; GFP-Nek2 (Supplementary Figure S1A). Cleaved caspase 3 and cleaved PARP levels were significantly decreased in shE2F3 compared to pLKO.1, and remained low in shE2F3; GFP and in shE2F3; GFP-Nek2, indicative of a lower extent of apoptotic response in all groups silenced for E2F3. No differences between levels of several pro-survival proteins, including Bcl-2, Bcl-xL, and Mcl-1 were found between control and shE2F3 or shE2F3 and shE2F3; GFP-Nek2. Proper temporal phosphorylation of histone 3 is required for chromosome condensation at mitosis and thus serves as a marker for mitotic cells [56]. Average levels of phospho-histone 3 were decreased in shE2F3 relative to pLKO.1, but did not reach significant levels; likewise, no significant differences were found when comparing shE2F3 to shE2F3; GFP-Nek2 or when comparing shE2F3; GFP to shE2F3; GFP-Nek2. Localization of histone H2AX phosphorylated at ser-139 (γ-H2AX) at DNA breaks is an effective measure of DNA breaks triggered by DNA damaging agents, oncogenes, or apoptosis [57–60] and no statistical significance was observed among groups. E-cadherin levels, a marker of cell adhesion, were only significantly reduced in shE2F3 tumors expressing GFP-Nek2.

### Nek2 overexpression in shE2F3 cells enhances formation of invasive protrusions

**Figure 3:**
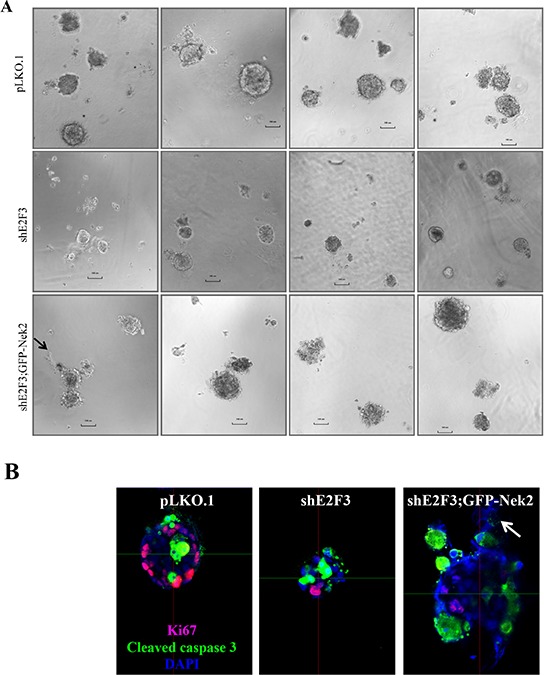

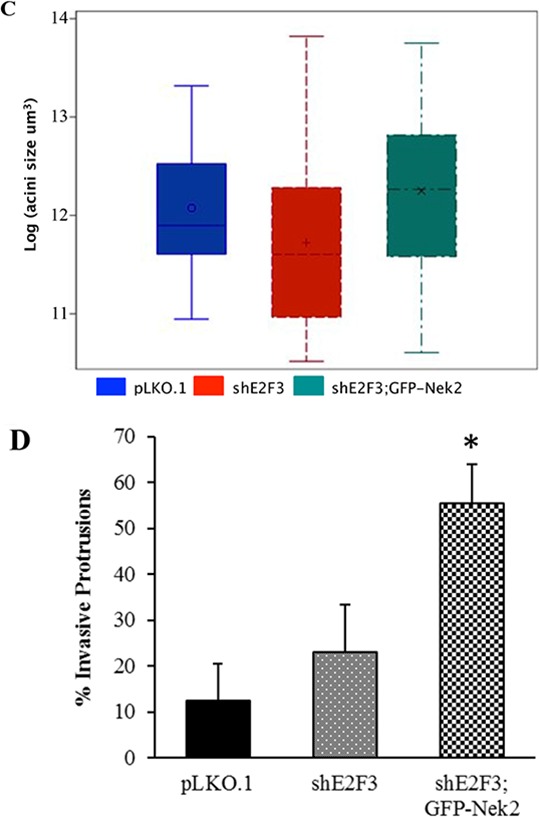

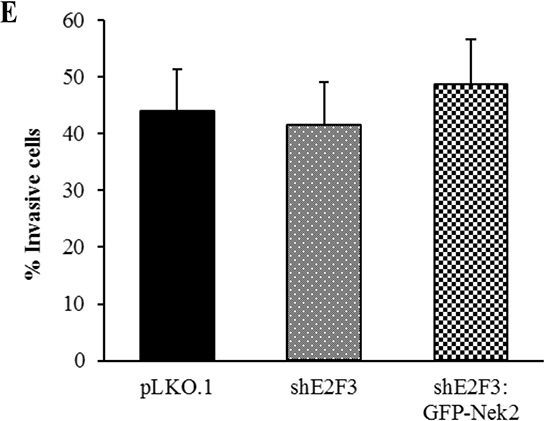
Nek2 overexpression in shE2F3 cells enhances formation of invasive protrusions **A.** Cells were grown in 3D matrigel culture for two weeks and acini were fixed in 4% paraformaldehyde and pictures of live acini were taken by Olympus IX51. **B.** Cells were processed for immuno fluorescence with the indicated markers to calculate. **C.** the volume of acini by using the Imaris software (log mean ± S.E.M) **D.** percentage of acini displaying protrusions was generated by manually scoring out cells detached from acini. **E.** The invasion assay was performed on cells by using BD Biocoat matrigel invasion chambers.

To address if there are any changes in the size and structure of three dimensional organoids, we plated cells, including vector control (pLKO.1), shE2F3 and shE2F3; GFP-Nek2 in reduced matrigel for 12 days until they formed acini [61]. Again, data presented here have to be interpreted conservatively, since we did not perform 3D cultures or invasion assays comparing shE2F3; GFP and shE2F3; GFP-Nek2. Visual examination suggested smaller acini in shE2F3 cells compared to controls and larger acini in shE2F3 cells overexpressing Nek2 (Figure 3A). The acini were processed for IF (Figure 3B) and their actual volume was measured using the Imaris software. Even though the average volume of shE2F3 group was smaller than that of pLKO.1, the results were not statistically significant (Figure 3C, Table 3).

**Table 3:** Volume of acini among groups

	Mean (Std. Err)(log)	*P*-value*	
pLKO.1	12.08 (0.18)	reference	
shE2F3	11.72 (0.22)	0.221	reference
shE2F3;GFP-Nek2	12.25 (0.18)	0.506	0.071

* Analysis of variance (ANOVA) was mainly used to estimate the mean and standard error of measurement in each cell line with a significant level of 0.05. The analysis was performed based on equal variance data assumption.

Recently, Godinho *et al* showed that invasive protrusions, a measure of cell invasion, were found in acinar structures derived from MCF10A cells overexpressing Plk4 that displayed CA and tetraploidy [62]. Thus, we addressed whether overexpression of Nek2 also induced invasive protrusions. We were able to identify acini with protrusions only in shE2F3 cells overexpressing Nek2 (Figure 3A and 3B, arrows). Therefore, we scored out percentages of invasive protrusions and found that more than 50% of shE2F3 cells overexpressing GFP-Nek2 displayed protrusions (Figure 3D). Based on the indication of invasiveness of shE2F3; GFP-Nek2 acini with protrusions, we next investigated if E2F3 knockdown or Nek2 overexpression in shE2F3 cells changed invasive ability in HCC1954 cells (Figure 3E). However, no statistical significant differences in the percentage invasive cells were found.

## DISCUSSION

To investigate the role of E2Fs in mouse mammary tumor growth, MMTV-ErbB2 mice knocked out for E2F1, E2F2 or E2F3 (E2F3 was conditionally deleted) were generated [17]. Knockout of E2F1 or E2F3 significantly delayed tumor onsets. Mechanistically, conditional knockout of E2F3 in mammary epithelial cells diminished the average percentage of cells within ERBB2 tumors undergoing S phase, but this deletion had no impact on tumor growth rates. Another relevant study demonstrated that knockout of E2F1, E2F2 or E2F3 reduced tumor onset driven by MMTV-ErbB2, with E2F1 or E2F3 ablation having no effects on tumor growth rates and ablation of E2F1 accelerating tumor growth [21]. However, there are no existing models showing that E2F silencing can stall or retard tumor growth of human breast cancers. To that end, we employed an orthotopic model of breast cancers using the Her2^+^ER^−^PR^−^ cell line HCC1954. This Her2^+^ model displays deregulated E2F1, E2F2 and E2F3 [18] and thus represents an environment in which lost E2F3 functions can be compensated by deregulated E2F1 and E2F2. Based on published literature thoroughly described in the introduction, we hypothesized that E2F3 knockdown reduced mammary tumor growth by modifying apoptosis, proliferation or mitosis. In this report, we demonstrate that stable silencing of a single E2F activator, E2F3, significantly reduces the growth rates and masses of highly malignant HCC1954 Her2^+^ tumors (Figure 1). Our previous study performed in cells cultured in monolayer showed that E2F3 knockdown suppressed CA/CIN, but did not affect DNA replication, entry into S phase or overall cycle progression in three Her2^+^ cells including HCC1954 [18]. Consistent with those results, E2F3 knockdown did not decrease the percentage of proliferating tumor cells relative to cells expressing vector control. This represents a major difference relative to the mouse MMTV-ERBB2 system, where knockout of E2F3 resulted in a 50% reduction of cells undergoing proliferation. Although shRNA resulted in an almost complete knockdown of E2F3 protein, differences between the mouse and human tumor systems may be due to the remaining E2F3 protein in cells expressing shE2F3 or differences in transcriptional targets between mouse and human mammary tumors.

Western blots and IF of xenograft samples showed apoptosis in control, pLKO.1 cells (Figure 2). E2F3 knockdown decreased the expression of markers of apoptosis (cleaved caspase-3 and cleaved PARP) and these decreased levels were maintained in all cell lines silenced for E2F3, including shE2F3; GFP, and shE2F3; GFP-Nek2. However, silencing E2F3 or overexpression of GFP-Nek2 did not change levels of protein of several regulators of survival, including Mcl1, Bcl-2 and Bcl-XL, or apoptosis, including Bax. Because regulation of apoptosis involves protein expression and complex phosphorylation events, future experiments in our laboratory will address how silencing of E2F3 suppresses apoptosis in Her2^+^ tumors. Overall, these data demonstrate that E2F3 knockdown decreases markers of apoptosis in HCC1954 tumors and that overexpression of GFP-Nek2 in shE2F3 cells does not modulate these percentages. While it can be argued that overexpression of GFP-Nek2 results in other types of cell death, live imaging performed in our laboratory in HCC1954 cells expressing shE2F3 or shE2F3; GFP-Nek2 did not find differences in the percentage of dying cells in these two groups over a 48 hour period [18].

While functional assays have shown the canonical role of E2F activators in proliferation, the implication of these proteins in mitosis is predicted by gene expression studies [8]. However, mitotic defects upon ablation of E2F activators have not been thoroughly studied. We showed that stable silencing of E2F3 severely decreased the percentage of HCC1954 cells undergoing cytokinesis, while the fraction of cells entering mitosis was unaffected [18]. The most novel finding of the present work is that silencing of E2F3 severely restricts the fraction of Her2^+^ tumor cells undergoing mitosis that strongly correlates with suppressed tumor growth. We postulate that restriction of mitosis is the primary mechanism suppressing tumor growth, since silencing E2F3 did not alter the percentage of tumor cells undergoing proliferation. Overall, our findings support the concept that targeting the mitotic machinery is an effective anti-tumor strategy [63, 64].

A major controversy in cancer biology is whether aneuploidy, which can be generated by multiple mechanisms, including CA, is transforming or tumor suppressive. While CA correlates with the aggressive behavior of triple negative breast cancers [42] and with the ability of breast tumors to metastasize [36], others find that increased aneuploidy is tumor suppressive [50, 65, 66]. We demonstrated that silencing Nek2 in several Her2^+^ cells reduce CA and CIN [43] and Nek2 overexpression rescues back CA in HCC1954 cells expressing shE2F3 in cell culture [18]. Nek2 regulates various mitotic functions, including centrosome separation at G2 phase [67], as well as the spindle assembly checkpoint [68, 69]. However, Nek2 kinase has other functions that may contribute to tumor growth, including phosphorylation of splicing factors that affect gene expression and cell cycle progression; in fact, knockdown of Nek2 or SRSF1 induces expression of pro-apoptotic variants from SRSF1-target genes and sensitizes cells to apoptosis [70]. Thus, we addressed whether rescuing back CA in tumors silenced for E2F3 influenced tumor growth. While GFP-Nek2 overexpression in shE2F3 cells did not modify tumor growth, an independent set of injections showed significant differences between shE2F3-GFP and GFP-Nek2, since GFP-Nek2 tumors were larger at early time points than shE2F3-GFP tumors (Figure 1). However, after day 10, there were no obvious differences in size between shE2F3; GFP and shE2F3; GFP-Nek2 and this is supported by observations that at the end of the experiment (day 21) tumor weights were similar. Western blots did not detect significant differences in levels of apoptosis effectors, mitotic or DNA damage markers between shE2F3 and shE2F3; GFP-Nek2 or between shE2F3; GFP and shE2F3; GFP-Nek2 (Figure 2 and Supplementary Figure S1). The only phenotypes affected by GFP-Nek2 expression in shE2F3 cells -detected by immunofluorescence of tumor sections- were increased CA and proliferation (Figure 2C), lower levels of E-Cadherin detected by western blots (Figure 2D and 2E) and the induction of invasive protrusions (Figure 3), suggestive of increased detachment of cells from acinar structures, one of the initial steps in the invasion process. This result is consistent to a similar observation in MCF10A cells that overexpress Plk4 mitotic kinase [62]. However, increased detachment triggered by GFP-Nek2 did not translate into increased invasion (Figure 3), suggesting that either HCC1954 cells have attained their maximal invasive potential, or that additional molecular alterations must cooperate with Nek2 to trigger full-fledged invasion. Another interpretation is that cancer pathways are already deregulated in HCC1954 cells and thus, further increases in CA and CIN do not affect fitness of these tumor cells. Nevertheless, additional models, including those overexpressing Nek2 in non-transformed mammary epithelial cells are needed to address whether the acquisition of CA and CIN influences tumor initiation, or contributes to other cancer-associated phenotypes, including invasion and metastasis.

## MATERIALS AND METHODS

### Cell lines

HCC1954 cells were purchased from ATCC (Manassas, Virginia) and maintained in 10% FBS supplemented RPMI1640 (R8758, Sigma, St. Louis, MO) and 2 μg/mL puromycin (Sigma) was added to HCC1954 pLKO.1 and shE2F3 cells. Lentiviral particles carrying shRNA against E2F3 were purchased from Open Biosystems (clone ID: TRCN0000013807). Both 2 μg/mL puromycin and 50 μg/mL hygromycin (H0654, Sigma) were added to grow HCC1954 shE2F3;GFP-Nek2 cells [18]. shE2F3 cells overexpressing GFP were generated as follows: the GFP expressing vector, pMONO-Hygro-GFP (Cat#pmonoh-GFP, InvivoGen, San Diego, CA) was transfected into shE2F3 cells and underwent hygromycin selection 48 hr after transfection. Pools of clones were harvested, GFP expression was confirmed and cells maintained in the same media as shE2F3;GFP-Nek2 cells.

### Cell preparation for mammary fat pad injection and measurement of tumor mass growth

A protocol was adapted from previously published work [46]. Briefly, 2 × 10^6^ cells were resuspended in 45 μL of RPMI1640 media (w/o FBS, w/o antibiotics) and 35 μL of Matrigel matrix (cat#354234, BD) was added to the cells and kept on ice until injection. Eight week old female athymic nude mice (Hsd:Athymic nude- Foxn1^nu^, Harlan, Indianapolis, IN) were injected in the right abdominal mammary glands (#9, from with pLKO.1 and in the hind, left mammary gland (#4) with shE2F3 cells (*n* = 8). For the second set (*n* = 5), mammary gland #9 was injected with shE2F3 cells and mammary gland #4 with shE2F3; GFP-Nek2 cells. For the third set (*n* = 4), shE2F3; GFP cells were injected in mammary gland #9 and shE2F3;GFP-Nek2 on #4. Tumor masses were tangible 1 week after injections and were measured 5–6 times/week for three weeks. Then, all mice were sacrificed; tumors were extirpated and split for paraffin blocks and protein extraction.

### Western blot analysis

Tumor mass was snap-frozen, pulverized, collected and resuspended in lysis buffer (50 mM HEPES, 250 mM KCl, 0.1 mM EDTA, 0.1 mM, 0.1% NP-40, 10% glycerol) as previously described [18]. Extracts were centrifuged at 12,000 rpm for 30 min at 4°C and supernatants were collected for protein quantitation. A total of 15 μg of total protein was analyzed by SDS-PAGE and blotted onto PVDF membranes to detect Bcl-2 (2870, Cell signaling), Mcl1 (5453, Cell signaling), Bcl-XL (2764, Cell Signaling, Danvers, MA), Bax (sc-493, Santa Cruz Biotechnology), FAK (sc-557, Santa-Cruz Biotechnology), cleaved caspase-3 (9661, Cell Signaling), cleaved PARP (5625, Cell Signaling), E-cadherin (610181, BD Biosciences, San Jose, CA), phospho-histone 3 (9713, Cell Signaling), and phospho γ-H2AX (9718, Cell Signaling). β-actin (4970, Cell Signaling) was used as a loading control. HRP-conjugated anti-mouse (sc-2005, Santa Cruz Biotechnology) or anti-rabbit (sc-2004, Santa Cruz biotechnology) were used as secondary antibodies and signals were detected using a Lumigen TMA-6 reagent (GE healthcare, Pittsburgh, PA).

### Invasion assay

The Invasion assay was performed according to the manufacturer's protocol (cat#354480, BD Biosciences, Bedford, MA). Briefly, 24-well invasion chambers (BD Biocoat Matrigel) were brought to room temperature and rehydrated by adding culture media. Three × 10^4^ cells/0.5 mL/insert were seeded in duplicates in either experimental or control inserts and 1 mL media was added to each well. Cells were incubated for 24 hours in a humidified, 5% CO_2_ 37°C incubator. Membranes were stained by crystal violet and pictures were taken by Zeiss Axioplan 2 microscope. Percent of invasion was generated by dividing mean number of cells invading through matrigel insert to the mean number of cell migrating through control insert membrane.

### Immunofluorescence

Paraffin blocks were made and cut in 5 μm sections for immunofluorescence analysis. Tumor sections were deparaffinized and antigen retrieval was performed by boiling sections for 3 min in pressurized cooker. Then, sections were blocked in 10% goat serum for 1–2 hours at room temperature, followed by primary antibodies (Ki67, VP-k452, vector laboratories, Burlingame, CA), E-cadherin (610181, BD Biosciences), cleaved caspase-3 (9661, Cell signaling), pericentrin (ab4448, Abcam, Cambridge, MA) and phospho histone 3 (9713, Cell signaling) overnight incubation at 4°C. Alexa Fluor 568-conjugated goat anti-rabbit (A21428, Invitrogen) or Alexa Fluor 555-conjugated goat anti-mouse antibody (A21422, Invitrogen, Carlsbad, CA) was used as a secondary, followed by 4′,6-diamidino-2-phenylindole(DAPI) counter staining. Images were acquired using Zeiss Axioplan 2 upright microscope. CellProfiler program [71] (Broad Institute, Cambridge, MA) was used to quantify staining; for cleaved caspase-3 and E-cadherin, red/blue ratio was calculated for each image, and percentage of positive cells were counted for Ki67 cells. For phospho-histone 3, positive cells were counted on the entire section and for pericentrin, ≥ 3 centrosomes/cell were counted from each image to generate CA percentages.

### 3D matrigel culture, measurement of acini volume and immunofluorescence of acini

The protocol was adapted from published work [61]. In brief, 8-well chamber slides (154534, Thermo Scientific, Waltham, MA) were coated with 60 μL of matrigel matrix (cat#354234, BD) and 2.5 × 10^3^ cells were resuspended in 2% matrigel and seeded in each well. Cells were cultured for 14 days while media was replenished twice per week. Acini were fixed by 4% paraformaldehyde for 20 minutes at room temperature. Before proceeding to cleaved caspase-3, E-cadherin, and Ki67 immunofluorescent staining, bright field pictures were taken. After washing three times with 1X PBS, acini, then, were permeabilized with 0.5% Triton X-100 prepared in 1X PBS for 10 minutes at 4°C, and washed three times (10–15 min each) with 1X PBS Glycine (1X PBS with 100 mM Glycine). Acini were blocked with 10% goat serum for 45–60 minutes at room temperature, followed by primary antibody incubation (cleaved caspase 3 and Ki67), diluted in 1% goat serum in 1X immunofluorescence buffer (IF) buffer overnight at 4°C. Acini were washed three times (20 min each) with IF buffer at room temperature, followed by secondary antibody (diluted in same buffer used in primary antibody incubation, either alexa Flour 555 goat anti-mouse (A-21422, Life technologies) for Ki67 and Alexa Fluor 488 donkey anti-rabbit (R37118, Life Technologies) for cleaved caspase and incubated for 50 minutes at room temperature. Acini were washed with 1x IF buffer for 20 minutes and DAPI (1 μg/mL) was applied as a counterstain. After washing with 1X PBS for 5 minutes at room temperature, slides were mounted with Prolong-Gold antifade (P36930, Life technologies) and pictures were taken with Zeiss LSM510 META confocal with Z-stack and the volume of immunostained acini were measured using Imaris software (Bitplane INC., South Windsor, CT).

### Statistical analysis

Unless otherwise stated, student *t*-test was applied to compare the differences between control and treated group and *p* value less than 0.05 was considered as significant. For tumor growth rates, the SAS statistical package v9.3 (SAS Institute, Inc., Cary, North Carolina) was used for analyses with a significant level of 0.05. Mixed effects model was implemented to estimate and compare the growth rate among three cell line groups. The correlation among the repeated measurements in each mouse over time as well as the correlation among the measurements on each side of mammary fat pad in the same mouse was accounted for as appropriate. The tumor volume was log transformed to meet the normality and equal variance assumption for the mixed effect model. Tumor weight was compared by both one-sided Wilcoxon Test, a nonparametric test, due to the small sample size. The significance level was set at 0.05. For the measurement of acini size, SAS was used for analyses with a significant level of 0.05. Analysis of variance (ANOVA) was mainly used to estimate the mean and standard error of measurement in each cell line. The data were first log transformed to meet the normality and equal variance data assumption for ANOVA.

## SUPPLEMENTARY FIGURE



## References

[1] Meraldi P, Lukas J, Fry AM, Bartek J, Nigg EA (1999). Centrosome duplication in mammalian somatic cells requires E2F and Cdk2- cyclin A. Nat Cell Biol.

[2] Trikha P, Sharma N, Opavsky R, Reyes A, Pena C, Ostrowski MC, Roussel MF, Leone G (2011). E2f1-3 are critical for myeloid development. J Biol Chem.

[3] Lee EY, Yuan TL, Danielian PS, West JC, Lees JA (2009). E2F4 cooperates with pRB in the development of extra-embryonic tissues. Developmental biology.

[4] Asp P, Acosta-Alvear D, Tsikitis M, van Oevelen C, Dynlacht BD (2009). E2f3b plays an essential role in myogenic differentiation through isoform-specific gene regulation. Genes Dev.

[5] Chong JL, Tsai SY, Sharma N, Opavsky R, Price R, Wu L, Fernandez SA, Leone G (2009). E2f3a and E2f3b contribute to the control of cell proliferation and mouse development. Mol Cell Biol.

[6] Hu T, Ghazaryan S, Sy C, Wiedmeyer C, Chang V, Wu L (2012). Concomitant inactivation of Rb and E2f8 in hematopoietic stem cells synergizes to induce severe anemia. Blood.

[7] Saavedra HI, Maiti B, Timmers C, Altura R, Tokuyama Y, Fukasawa K, Leone G (2003). Inactivation of E2F3 results in centrosome amplification. Cancer Cell.

[8] Ishida S, Huang E, Zuzan H, Spang R, Leone G, West M, Nevins JR (2001). Role for E2F in control of both DNA replication and mitotic functions as revealed from DNA microarray analysis. Mol Cell Biol.

[9] Nevins JR (2001). The Rb/E2F pathway and cancer. Hum Mol Genet.

[10] Nevins JR, Leone G, DeGregori J, Jakoi L (1997). Role of the Rb/E2F pathway in cell growth control. J Cell Physiol.

[11] Cooper CS, Nicholson AG, Foster C, Dodson A, Edwards S, Fletcher A, Roe T, Clark J, Joshi A, Norman A, Feber A, Lin D, Gao Y, Shipley J, Cheng SJ (2006). Nuclear overexpression of the E2F3 transcription factor in human lung cancer. Lung Cancer.

[12] Foster CS, Falconer A, Dodson AR, Norman AR, Dennis N, Fletcher A, Southgate C, Dowe A, Dearnaley D, Jhavar S, Eeles R, Feber A, Cooper CS (2004). Transcription factor E2F3 overexpressed in prostate cancer independently predicts clinical outcome. Oncogene.

[13] Reimer D, Hubalek M, Kiefel H, Riedle S, Skvortsov S, Erdel M, Hofstetter G, Concin N, Fiegl H, Muller-Holzner E, Marth C, Altevogt P, Zeimet AG (2011). Regulation of transcription factor E2F3a and its clinical relevance in ovarian cancer. Oncogene.

[14] Wu Q, Hoffmann MJ, Hartmann FH, Schulz WA (2005). Amplification and overexpression of the ID4 gene at 6p22.3 in bladder cancer. Mol Cancer.

[15] De Meyer T, Bijsmans IT, Van de Vijver KK, Bekaert S, Oosting J, Van Criekinge W, van Engeland M, Sieben NL (2009). E2Fs mediate a fundamental cell-cycle deregulation in high-grade serous ovarian carcinomas. The Journal of pathology.

[16] Andre F, Job B, Dessen P, Tordai A, Michiels S, Liedtke C, Richon C, Yan K, Wang B, Vassal G, Delaloge S, Hortobagyi GN, Symmans WF, Lazar V, Pusztai L (2009). Molecular characterization of breast cancer with high-resolution oligonucleotide comparative genomic hybridization array. Clin Cancer Res.

[17] Wu L, de Bruin A, Wang H, Simmons T, Cleghorn W, Goldenberg LE, Sites E, Sandy A, Trimboli A, Fernandez SA, Eng C, Shapiro C, Leone G (2013). Selective roles of E2Fs for ErbB2- and Myc-mediated mammary tumorigenesis. Oncogene.

[18] Lee MY, Moreno CS, Saavedra HI (2014). The E2F activators signal and maintain centrosome amplification in breast cancer cells. Mol Cell Biol.

[19] Baldini E, Camerini A, Sgambato A, Prochilo T, Capodanno A, Pasqualetti F, Orlandini C, Resta L, Bevilacqua G, Collecchi P (2006). Cyclin A and E2F1 overexpression correlate with reduced disease-free survival in node-negative breast cancer patients. Anticancer Res.

[20] Fujiwara K, Yuwanita I, Hollern DP, Andrechek ER (2011). Prediction and genetic demonstration of a role for activator E2Fs in Myc-induced tumors. Cancer Res.

[21] Andrechek ER (2013). HER2/Neu tumorigenesis and metastasis is regulated by E2F activator transcription factors. Oncogene.

[22] Sorlie T, Perou CM, Tibshirani R, Aas T, Geisler S, Johnsen H, Hastie T, Eisen MB, van de Rijn M, Jeffrey SS, Thorsen T, Quist H, Matese JC, Brown PO, Botstein D, Eystein Lonning P (2001). Gene expression patterns of breast carcinomas distinguish tumor subclasses with clinical implications. Proc Natl Acad Sci U S A.

[23] Lukas J, Petersen BO, Holm K, Bartek J, Helin K (1996). Deregulated expression of E2F family members induces S-phase entry and overcomes p16INK4A-mediated growth suppression. Mol Cell Biol.

[24] Johnson DG, Schwarz JK, Cress WD, Nevins JR (1993). Expression of transcription factor E2F1 induces quiescent cells to enter S phase. Nature.

[25] Leone G, DeGregori J, Yan Z, Jakoi L, Ishida S, Williams RS, Nevins JR (1998). E2F3 activity is regulated during the cell cycle and is required for the induction of S phase. Genes Dev.

[26] Humbert PO, Verona R, Trimarchi JM, Rogers C, Dandapani S, Lees JA (2000). E2f3 is critical for normal cellular proliferation. Genes Dev.

[27] Wu L, Timmers C, Maiti B, Saavedra HI, Sang L, Chong GT, Nuckolls F, Giangrande P, Wright FA, Field SJ, Greenberg ME, Orkin S, Nevins JR, Robinson ML, Leone G (2001). The E2F1-3 transcription factors are essential for cellular proliferation. Nature.

[28] Polager S, Kalma Y, Berkovich E, Ginsberg D (2002). E2Fs up-regulate expression of genes involved in DNA replication, DNA repair and mitosis. Oncogene.

[29] He L, Yang H, Ma Y, Pledger WJ, Cress WD, Cheng JQ (2008). Identification of Aurora-A as a direct target of E2F3 during G2/M cell cycle progression. J Biol Chem.

[30] Tategu M, Nakagawa H, Sasaki K, Yamauchi R, Sekimachi S, Suita Y, Watanabe N, Yoshid K (2008). Transcriptional regulation of human polo-like kinases and early mitotic inhibitor. J Genet Genomics.

[31] Harrison MK, Adon AM, Saavedra HI (2011). The G1 phase Cdks regulate the centrosome cycle and mediate oncogene-dependent centrosome amplification. Cell division.

[32] Roylance R, Endesfelder D, Gorman P, Burrell RA, Sander J, Tomlinson I, Hanby AM, Speirs V, Richardson AL, Birkbak NJ, Eklund AC, Downward J, Kschischo M, Szallasi Z, Swanton C (2011). Relationship of extreme chromosomal instability with long-term survival in a retrospective analysis of primary breast cancer. Cancer epidemiology, biomarkers & prevention : a publication of the American Association for Cancer Research, cosponsored by the American Society of Preventive Oncology.

[33] Fukasawa K (2007). Oncogenes and tumour suppressors take on centrosomes. Nature reviews Cancer.

[34] Lingle WL, Lutz WH, Ingle JN, Maihle NJ, Salisbury JL (1998). Centrosome hypertrophy in human breast tumors: implications for genomic stability and cell polarity. Proc Natl Acad Sci U S A.

[35] Guo HQ, Gao M, Ma J, Xiao T, Zhao LL, Gao Y, Pan QJ (2007). Analysis of the cellular centrosome in fine-needle aspirations of the breast. Breast Cancer Res.

[36] Schneeweiss A, Sinn HP, Ehemann V, Khbeis T, Neben K, Krause U, Ho AD, Bastert G, Kramer A (2003). Centrosomal aberrations in primary invasive breast cancer are associated with nodal status and hormone receptor expression. Int J Cancer.

[37] Zeng X, Shaikh FY, Harrison MK, Adon AM, Trimboli AJ, Carroll KA, Sharma N, Timmers C, Chodosh LA, Leone G, Saavedra HI (2010). The Ras oncogene signals centrosome amplification in mammary epithelial cells through cyclin D1/Cdk4 and Nek2. Oncogene.

[38] Lewis WE (1990). Prognostic significance of flow cytometric DNA analysis in node-negative breast cancer patients. Cancer.

[39] Visscher DW, Zarbo RJ, Jacobsen G, Kambouris A, Talpos G, Sakr W, Crissman JD (1990). Multiparametric deoxyribonucleic acid and cell cycle analysis of breast carcinomas by flow cytometry. Clinicopathologic correlations. Lab Invest.

[40] Dowle CS, Owainati A, Robins A, Burns K, Ellis IO, Elston CW, Blamey RW (1987). Prognostic significance of the DNA content of human breast cancer. Br J Surg.

[41] Smith FB, Zappi ME (1993). Relationships between image cytometric DNA index, proliferation fraction and multiploidy and conventional nuclear grade in breast carcinoma. Modern pathology : an official journal of the United States and Canadian Academy of Pathology, Inc.

[42] Pannu V, Mittal K, Cantuaria G, Reid MD, Li X, Donthamsetty S, McBride M, Klimov S, Osan R, Gupta MV, Rida PC, Aneja R (2015). Rampant centrosome amplification underlies more aggressive disease course of triple negative breast cancers. Oncotarget.

[43] Harrison Pitner MK, Saavedra HI (2013). Cdk4 and nek2 signal binucleation and centrosome amplification in a her2+ breast cancer model. PLoS One.

[44] Saavedra HI, Wu L, de Bruin A, Timmers C, Rosol TJ, Weinstein M, Robinson ML, Leone G (2002). Specificity of E2F1, E2F2, and E2F3 in mediating phenotypes induced by loss of Rb. Cell Growth Differ.

[45] Putzer BM (2007). E2F1 death pathways as targets for cancer therapy. J Cell Mol Med.

[46] Shim JS, Rao R, Beebe K, Neckers L, Han I, Nahta R, Liu JO (2012). Selective inhibition of HER2-positive breast cancer cells by the HIV protease inhibitor nelfinavir. J Natl Cancer Inst.

[47] Marina M, Saavedra HI (2014). Nek2 and Plk4: prognostic markers, drivers of breast tumorigenesis and drug resistance. Front Biosci (Landmark Ed).

[48] Hagen KR, Zeng X, Lee M-Y, Tucker Kahn S, Harrison Pitner MK, Zaky SS, Liu Y, O'Regan RM, Deng X, Saavedra HI (2013). Silencing CDK4 radiosensitizes breast cancer cells by promoting apoptosis. Cell division.

[49] Ogden A, Rida PC, Aneja R (2013). Heading off with the herd: how cancer cells might maneuver supernumerary centrosomes for directional migration. Cancer metastasis reviews.

[50] Weaver BA, Silk AD, Cleveland DW (2008). Low rates of aneuploidy promote tumorigenesis while high rates of aneuploidy cause cell death and tumor suppression. Cell Oncol.

[51] Godinho SA, Kwon M, Pellman D (2009). Centrosomes and cancer: how cancer cells divide with too many centrosomes. Cancer metastasis reviews.

[52] Neve RM, Chin K, Fridlyand J, Yeh J, Baehner FL, Fevr T, Clark L, Bayani N, Coppe JP, Tong F, Speed T, Spellman PT, DeVries S, Lapuk A, Wang NJ, Kuo WL (2006). A collection of breast cancer cell lines for the study of functionally distinct cancer subtypes. Cancer Cell.

[53] Krzywicka-Racka A, Sluder G (2011). Repeated cleavage failure does not establish centrosome amplification in untransformed human cells. J Cell Biol.

[54] Fukasawa K (2008). p53, cyclin-dependent kinase and abnormal amplification of centrosomes. Biochim Biophys Acta.

[55] Youle RJ, Strasser A (2008). The BCL-2 protein family: opposing activities that mediate cell death. Nat Rev Mol Cell Biol.

[56] Goto H, Tomono Y, Ajiro K, Kosako H, Fujita M, Sakurai M, Okawa K, Iwamatsu A, Okigaki T, Takahashi T, Inagaki M (1999). Identification of a novel phosphorylation site ∫on histone H3 coupled with mitotic chromosome condensation. J Biol Chem.

[57] Ray S, Atkuri KR, Deb-Basu D, Adler AS, Chang HY, Herzenberg LA, Felsher DW (2006). MYC can induce DNA breaks *in vivo* and *in vitro* independent of reactive oxygen species. Cancer Res.

[58] Rogakou EP, Boon C, Redon C, Bonner WM (1999). Megabase chromatin domains involved in DNA double-strand breaks *in vivo*. J Cell Biol.

[59] Rogakou EP, Pilch DR, Orr AH, Ivanova VS, Bonner WM (1998). DNA double-stranded breaks induce histone H2AX phosphorylation on serine 139. J Biol Chem.

[60] Rogakou EP, Nieves-Neira W, Boon C, Pommier Y, Bonner WM (2000). Initiation of DNA fragmentation during apoptosis induces phosphorylation of H2AX histone at serine 139. J Biol Chem.

[61] Debnath J, Muthuswamy SK, Brugge JS (2003). Morphogenesis and oncogenesis of MCF-10A mammary epithelial acini grown in three-dimensional basement membrane cultures. Methods.

[62] Godinho SA, Picone R, Burute M, Dagher R, Su Y, Leung CT, Polyak K, Brugge JS, Thery M, Pellman D (2014). Oncogene-like induction of cellular invasion from centrosome amplification. Nature.

[63] Sudakin V, Yen TJ (2007). Targeting mitosis for anti-cancer therapy. BioDrugs : clinical immunotherapeutics, biopharmaceuticals and gene therapy.

[64] Schmit TL, Ahmad N (2007). Regulation of mitosis via mitotic kinases: new opportunities for cancer management. Mol Cancer Ther.

[65] Weaver BA, Cleveland DW (2007). Aneuploidy: instigator and inhibitor of tumorigenesis. Cancer Res.

[66] Weaver BA, Silk AD, Montagna C, Verdier-Pinard P, Cleveland DW (2007). Aneuploidy acts both oncogenically and as a tumor suppressor. Cancer Cell.

[67] Mardin BR, Lange C, Baxter JE, Hardy T, Scholz SR, Fry AM, Schiebel E (2010). Components of the Hippo pathway cooperate with Nek2 kinase to regulate centrosome disjunction. Nat Cell Biol.

[68] Wei R, Ngo B, Wu G, Lee WH (2011). Phosphorylation of the Ndc80 complex protein, HEC1, by Nek2 kinase modulates chromosome alignment and signaling of the spindle assembly checkpoint. Molecular biology of the cell.

[69] Liu Q, Hirohashi Y, Du X, Greene MI, Wang Q (2010). Nek2 targets the mitotic checkpoint proteins Mad2 and Cdc20: a mechanism for aneuploidy in cancer. Experimental and molecular pathology.

[70] Naro C, Barbagallo F, Chieffi P, Bourgeois CF, Paronetto MP, Sette C (2014). The centrosomal kinase NEK2 is a novel splicing factor kinase involved in cell survival. Nucleic Acids Res.

[71] Kamentsky L, Jones TR, Fraser A, Bray MA, Logan DJ, Madden KL, Ljosa V, Rueden C, Eliceiri KW, Carpenter AE (2011). Improved structure, function and compatibility for CellProfiler: modular high-throughput image analysis software. Bioinformatics.

