# Supplementary material for: Silencing of E2F3 suppresses tumor growth of Her2+ breast cancer cells by restricting mitosis
Source: Oncotarget. 2015 Oct 26;6(35):37316–34. doi: 10.18632/oncotarget.5686 (PMC4741932; doi:10.18632/oncotarget.5686)
Supplement: Supplementary file 1 [file oncotarget-06-37316-s001.pdf]

SUPPLEMENTARY FIGURE

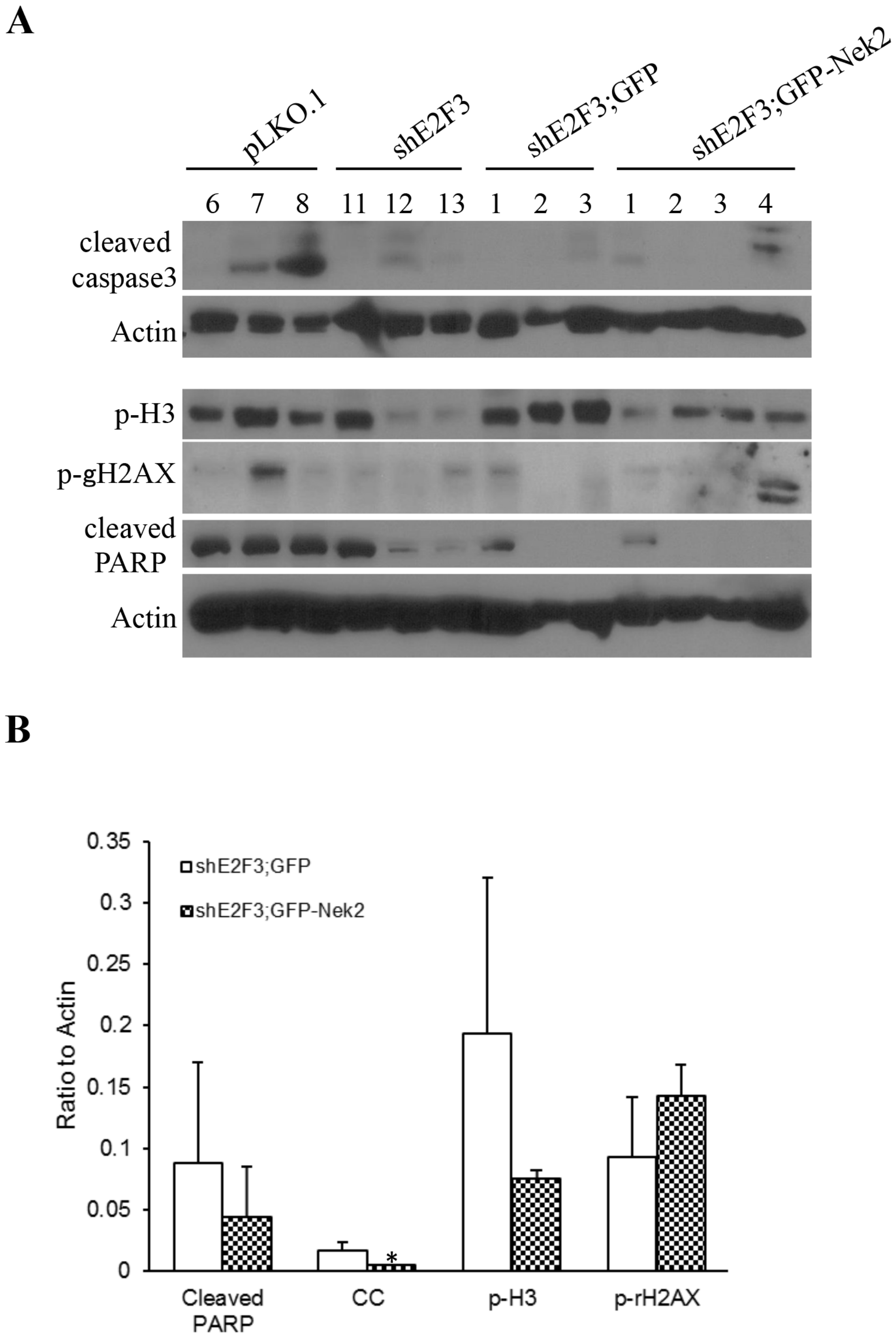

Supplementary Figure S1: Generation of shE2F3;GFP cells and measurement of tumor growth. A. An independent Western blot was performed in three new tumor samples of the pLKO.1 vs. shE2F3 group, and new tumors from the shE2F3; GFP vs. shE2F3;GFP-Nek2 groups. **B.** quantification of protein levels.
